# The cumulative impact of seizures: the science underlying how seizures beget seizures

**DOI:** 10.3389/fneur.2026.1793970

**Published:** 2026-05-20

**Authors:** Richard M. Gustin, Taha Gholipour, Jurriaan M. Peters

**Affiliations:** 1 Neurelis, Inc., San Diego, CA, United States; 2 Department of Neurosciences, University of California San Diego, La Jolla, CA, United States; 3 Department of Neurology, Division of Epilepsy and Neurophysiology, Boston Children's Hospital and Harvard Medical School, Boston, MA, United States

**Keywords:** drug-resistant epilepsy, epilepsy treatment, hyperexcitability, quality of life, seizure progression, seizure timeline

## Abstract

Seizures are symptoms of epilepsy but spontaneous seizure recurrence can also be considered a biomarker of disease progression. The temporary imbalance between excitatory and inhibitory drive that culminates in a hyperexcitable, hypersynchronous state clinically observed as seizure, initiates an insidious cascade of neurochemical, structural, genetic, epigenetic, and neuroinflammatory processes that increases seizure frequency, duration, and severity. As seizure activity increases, the hyperexcitable and hypersynchronous states entrain neuronal networks, leading to a further reduction in seizure threshold and further increases in seizure frequency, duration, and severity over time. The pathological circuitry generated and sustained by epileptic events not only drive hyperexcitable states within the seizure circuit but also can affect wider network connections. These effects lead to altered network physiology, which is associated with neuropsychiatric comorbidities, such as cognitive decline and psychiatric disorders, and can impact the operation of other organ systems. Here, we review the current understanding of how seizures can hijack normal brain function and set off a cascade of pathobiological events that support continued seizure activity and epilepsy progression. Understanding the neurobiology of seizure progression is fundamental to building comprehensive treatment strategies and developing new pharmacotherapies that aim to retrain seizure circuitry, with the goal of reducing overall seizure burden, improving quality of life, and limiting disease progression.

## 1 Introduction

In the late 19th century, Sir William Gowers described a phenomenon in some patients with epilepsy as “seizures beget seizures” (1). That is, epilepsy and its seizure burden seem to progress over time. What evidence do we have today for that observation? Kwan and Brodie reported the rate of drug-resistant epilepsy (DRE) in newly diagnosed patients with epilepsy (2). Their seminal research demonstrated that 64% of patients were able to become seizure free for a minimum of 12 months on a regimen of antiseizure medications (ASMs), while the remaining patients continued to have seizures despite receiving treatment (2). Unfortunately, even with over 30 ASMs available today, the rate of DRE has not changed since that initial report (3). In fact, if seizure freedom is not achieved with a single ASM, the likelihood of achieving seizure freedom further declines with each additional ASM (2, 3). In this article, we review the cascade of events that occur after an initial seizure and how recurrent seizures lead to structural and functional changes that promote seizure-generating networks in patients with DRE. We review the dynamic processes that drive the pathological circuitry underlying DRE that may be clinically leveraged to improve patient outcomes.

Sustained structural changes and aging nomograms suggest that the cumulative impact of seizures leads to neuronal injury and is detrimental to brain health, even in the absence of detectable permanent brain damage from a single seizure event (e.g., status epilepticus) (4, 5). Although seizure freedom remains unattainable for many patients with DRE, what insights from decades of scientific research can inform clinical treatment strategies to reduce seizure burden, improve patient outcomes, and improve quality of life (QoL) for those living with DRE? In addition to optimizing ASM regimen, effective acute treatment of seizure episodes with immediate-use seizure medications, implantable neuromodulation devices, or surgery can limit total seizure activity, but for many patients seizure recurrence remains an issue (6–9). By understanding the neurobiology underlying seizure progression, we may be able to better understand how treatments can be used or developed to retain pathological seizure circuits and restore normal function. We will explore how this approach may improve patient outcomes, after reviewing the neural processes following an initial seizure and the cascade of events that leads to DRE.

## 2 Methods

The authors conducted comprehensive literature searches in PubMed for peer-reviewed scientific and medical literature on seizure and epilepsy published through 2025, using the following search terms: “hyperexcitability,” “hypersynchronization,” “neuroinflammation,” “extracellular matrix,” “genetic and epigenetic factors,” “structural and functional changes,” “cell death,” “quality of life,” “cognitive impairment,” “mental health,” and “mortality and morbidity.” More refined searches expanding each topic were performed as needed, and the bibliographies of relevant articles were searched as well. Literature was included if it was published in a peer-reviewed journal, published in English, and focused on the topics of seizure, epilepsy, and neuronal mechanism. Literature was excluded if it consisted of only abstracts or editorials lacking any peer-reviewed referenced studies. Titles and abstracts were evaluated for relevance, followed by a full-text review of the relevant articles. A qualitative analysis of the selected articles was carried out to identify areas of consensus and contradiction in the current research, and to summarize the current understanding of the neurobiology underlying seizure progression across animal and clinical studies. The information collected in the review was synthesized into a timeline that most accurately reflected the cascade of neurobiological events that occur following a seizure. This review article is based on previously published studies and does not present any new or original data generated or collected by the authors. All data cited in this review article are available in the public domain and sourced from the works cited in the References section. Figures in this review were created using BioRender, a scientific image and illustration software.

## 3 Transition from normal function to seizure generation

Before reviewing the neurobiology underlying seizure progression, the canonical neuronal mechanisms that drive normal behavior and learning and memory must be considered. This provides a foundational understanding of how hyperexcitable and hypersynchronous states are reinforced, leading to increased seizure burden. This is not intended to be a comprehensive review of neuronal plasticity and learning and memory; however, there are several critically conserved mechanisms that should be discussed prior to examining the mechanisms underlying seizure progression.

The Neurophysiological Postulate, posed by Hebb in 1949, is a fundamental principle of neuroscience that influenced our understanding of how activity-dependent molecular and morphological changes in the brain are critical for normal development, information processing and storage, and learning and memory (10–12). The idea that “neurons that fire together, wire together,” is the foundation for what we know today as use-dependent plasticity (10–12).

**Figure 1:**
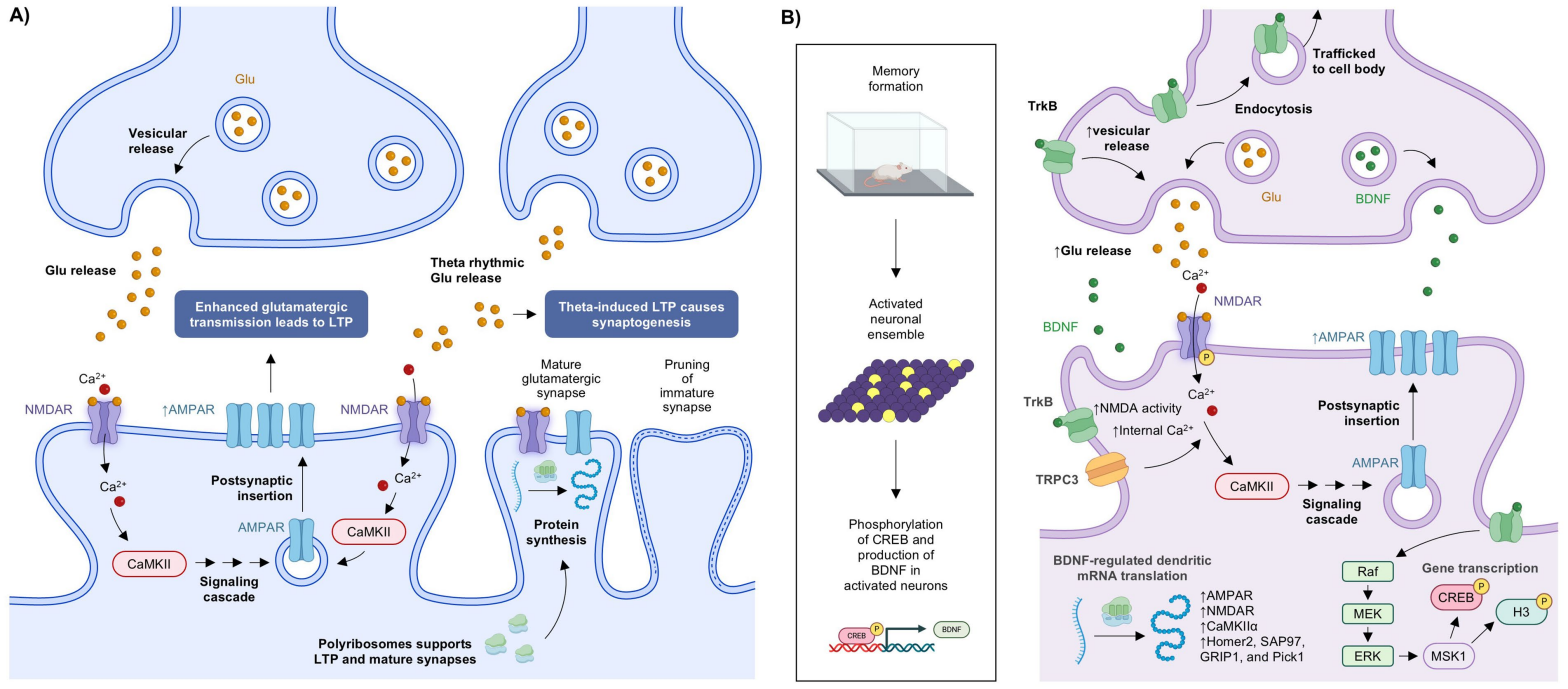
Long-term potentiation (LTP) and memory formation. Neural mechanisms underlying LTP **(A)** and memory formation **(B)**. Figure created in BioRender. AMPAR, *α*-amino-3-hydroxy-5-methyl-4-isoxazolepropionic acid receptor; BDNF, brain-derived neurotrophic factor; Ca^2+^, calcium ion; CaMKII, calcium/calmodulin-dependent protein kinase II; CREB, cAMP-responsive element–binding protein; ERK, extracellular signal-regulated kinase; Glu, glutamate; GRIP1, glutamate receptor interacting protein 1; H3, histone H3; Homer2, homer scaffold protein 2; MEK, mitogen-activated protein kinase kinase; mRNA, messenger RNA; MSK1, mitogen- and stress-activated protein kinase 1; NMDAR, *N*-methyl-D-aspartate receptor; P, phosphorylation; Pick1, protein interacting with C kinase 1; Raf, rapidly accelerated fibrosarcoma; RNA, ribonucleic acid; SAP97 synapse-associated protein 97; TrkB, tropomyosin receptor kinase B; TRPC3, transient receptor potential cation channel subfamily C member 3.

How can a brain that is predisposed to seizure (i.e., hyperexcitability and hypersynchronization) initiate and propagate epileptiform activity? According to the Neurophysiologic Postulate, a cascade of events begins as transient fluctuations transitioning to sustained long-term changes that drive a pattern of activity across a neuronal ensemble, or groups of neurons exhibiting coordinated activity in a recurrent fashion that are broadly distributed across the brain, which connect brain regions and drive learning, memory, and behavior (12, 13). Figure 1 briefly reviews long-term potentiation (LTP) and memory formation.

The structural and functional cellular correlates of learning and memory can be thought of as the neural synapse and LTP, respectively. The synapse is composed of the pre- and postsynaptic neurons, and the strengthening of their connection can be recorded as LTP, which is critical to the dynamic functioning of neuronal circuitry. The postsynaptic density (PSD) of an excitatory neuron actively engages in the learning process. The activation of postsynaptic neurons leads to a transient increase in postsynaptic Ca^2+^ (Figure 1A) (13, 14). Ca^2+^/calmodulin-dependent protein kinase II (CaMKII), along with additional signaling pathways, integrate these localized Ca^2+^ transients to induce a cascade of downstream effects in the dendritic spines, including enhanced trafficking of *α*-aminio-3-hydroxy-5-methyl-4-isoxazolepropoinic acid (AMPA) and N-methyl-D-aspartate (NMDA) receptors, thus increasing glutamatergic transmission (13–16). The strengthening of these neuronal connections is archived by the longer-lasting morphological changes in these excitatory synapses (13, 17). This structural synaptic plasticity is a fundamental feature of LTP. Following theta-burst induction of LTP, there is an increased ratio of excitatory/inhibitory (E/I) synapses that emerges within the first 30 min, which continues to increase over several hours (18). Additionally, within the first 30 min after LTP induction, there is an increase in synaptogenesis leading to an increase in mature synapses, and by 2 h after LTP induction, there is pruning of immature spines (18). Protein synthesis is required to maintain LTP, as polyribosomes are trafficked from dendritic shafts into the dendritic spines in larger PSDs and are associated with mature synapses (18). These data are consistent with learning and memory models (i.e., water maze, passive avoidance, contextual fear conditioning, spatial training, and trace eyeblink conditioning) that demonstrate a transient increase in synaptogenesis within the first 6 h after training, proceeded by an increase in PSD volume 24 h after training (17). Taken together, the available evidence indicates that increased neuronal activity leads to significant changes in synaptic morphology that impact how the involved neuronal ensemble or neuronal circuits function, leading to increased neuronal firing frequency and reduced latency to fire (17, 19).

These molecular and structural changes also induce local transcriptional changes, which strengthen the connectivity within the neuronal ensemble to reinforce and maintain activity throughout the circuit (13). Central to the regulation of synaptic transmission, LTP, synaptic strengthening, and memory formation is the function of brain-derived neurotrophic factor (BDNF) (20). During the development of memories, the transcription factor cyclic AMP response element–binding protein (CREB) is selectively expressed in neurons of the activated ensemble (Figure 1B) (13, 16). The phosphorylation and expression of CREB increases within the first 15 min of neuronal activation, and subsequently increases BDNF expression in these neurons within hours after activation (12). BDNF plays a number of roles within both the pre- and postsynaptic neurons, from short-term effects induced by BDNF protein phosphorylation at the synapse to long-term regulation of messenger RNA (mRNA) translation at the synapse and changes in transcription (20).

The role of BDNF in synaptic plasticity was reviewed by Leal et al. (20). In brief, phosphorylation of BDNF increases binding and activation of tropomyosin-related kinase B (TrkB) receptors (20, 21). At the presynaptic terminal, BDNF activation of TrkB receptors increases synaptic transmission by trafficking synaptic vesicles to the active zone for synaptic release (20). In addition, the BDNF/TrkB complex is internalized and trafficked to the cell body, where it is able to alter gene expression and mRNA translation (20). Postsynaptically, BDNF activation of TrkB receptors leads to phosphorylation of NMDA receptors, increasing NMDA receptor activity and synaptic transmission, in addition to promoting the release of internal Ca^2+^ stores and activation of transient receptor potential cation channel subfamily C member 3 (TRPC3), which increase postsynaptic Ca^2+^ concentrations, thus activating CaMKII and enhancing glutamatergic signaling through AMPA and NMDA receptor trafficking (14–16, 20). Along with playing a role in the postsynaptic signaling cascades, BDNF also regulates dendritic mRNA translation at free polysomes and endoplasmic reticulum–associated ribosomes (20). Local translation of mRNA is critical to synaptic regulation, with an estimated 2,550 mRNAs coding for proteins present in the dendrites and axons, from multiple protein families (22). This includes rapid upregulation of AMPA receptors, NMDA receptors, CaMKIIα, and the PSD scaffolding proteins homer scaffold protein 2 (Homer2), synapse-associated protein 97 (SAP97), glutamate receptor interacting protein 1 (GRIP1), and protein interacting with C kinase 1 (Pick1) within dendrites, promoting synaptic transmission and synaptic strengthening (23–27).

The repetitive stimulation and activation of neurochemical and second messenger signaling pathways by learning behavior lead to longer-lasting changes within activated neurons. Sustained changes and long-term synaptic enhancement are driven by the coupling of neuronal excitability to gene transcription (16). These activity-dependent acute changes stimulate longer-term changes at the epigenetic level that maintain the strength and connectivity of the neuronal ensemble (13, 18). Long-term regulation of neuronal activity and morphology involves homeostatic synaptic plasticity, which is the mechanism that controls synaptic communication (28). This synaptic scaling provides balance between synaptic excitability and inhibition across all synapses, thus providing a mechanism to prevent unconstrained activity and the flexibility to establish new homeostatic set points depending on the E/I activity experienced (28, 29). Critical to this long-term regulation is the role of mitogen- and stress-activated protein kinase 1 (MSK1), which following activity-dependent stimulation, facilitates longitudinal changes in synaptic structure and function, gene expression, and learning and memory (30). MSK1 is activated by BDNF and able to translate activity-dependent stimulation into enduring cellular, molecular, and genetic effects through phosphorylation of CREB and histone H3, thus regulating gene transcription and leading to both enhanced LTP and long-term depression (LTD) (16, 28–31). MSK1 regulates expression of an array of genes and proteins, including (1) the AMPA receptor subunit GluA1; (2) AMPA receptor trafficking protein; (3) activity-regulated cytoskeleton-associated protein (Arc), an AMPA receptor binding protein; (4) the immediate early genes *FOS* and *Pick1*; (5) the scaffold proteins PSD-95 and PSD-93; (6) the transcription factors Erg1 and FOS; (7) CaMKII; (8) the cytokine tumor necrosis factor alpha (TNFα); and (9) BDNF (through a positive feedback loop). All of these play a role in homeostatic and activity-dependent plasticity, allowing for an increased synaptic dynamic range and enhanced information processing (28–31). Although this review focuses primarily on the roles of BDNF and CREB due to their involvement in seizure propagation, there are many other receptor systems and signaling pathways that contribute to LTP, LTD, homeostatic plasticity, and learning and memory, including multiple signaling cascades that lead to differential CREB phosphorylation (31).

Finally, long-term memory conservation and preservation can be achieved through epigenetic regulation, encoded by differential DNA methylation and histone modifications that are passed on from one generation to the next (32). This encoding allows for appropriate and sustained transcriptional regulation that underlies functional changes associated learning and memory (32). Guan et al. posit that these cellular memory events within individual neurons may translate to the larger neuronal ensemble, and this is the mechanism by which sensory information is stored in these neuronal circuits (32).

## 4 Seizure and maladaptive reinforcement of brain circuits

The brain’s structural-functional relationship can also be hijacked by an epileptogenic mechanism, leading to a maladaptive reinforcement of a propagation or reverberating circuit (13). Homeostatic imbalance from repeated seizures alter synaptic structure and function, and continued activation reinforces the hyperexcitable state that predisposes the neuronal circuitry to additional seizure activity.

Serial imaging studies have demonstrated the long-term impact of continued seizure over time at the individual and group levels (33). Jackson et al. described a series of changes in new-onset temporal lobe epilepsy (TLE) that began with a transient and isolated increase in T2 hyperintensity in the neuronal layer of the hippocampus on initial magnetic resonance imaging (MRI), followed eight months later by MRI evidence of unilateral hippocampal sclerosis with loss of hippocampal internal architecture and abnormalities in both T2- and T1-weighted sequences, signaling gliosis and cell loss (34). The FEBSTAT (febrile status epilepticus) study also demonstrated how an initial seizure event can have long-term negative effects on brain development (35). Following an episode of complex febrile seizures with status epilepticus, acute hippocampal injury, as evidenced by an abnormal T2 signal, was noted in 11.5% of patients (*n* = 22/191), which was not seen in any of the control patients (*n* = 96) (35). Extrahippocampal abnormalities were also more frequent in the patients with febrile status epilepticus (temporal level, amygdala, and insula), suggesting more extensive brain injury that may impact further progression and propagation of seizures and the development of comorbidities (35).

### 4.1 Acute seizure: hyperexcitability and hypersynchronization

A seizure reflects hyperexcitability and hypersynchronization driven by E/I imbalance (36). Whether due to a preexisting vulnerability or seizure-induced plasticity, seizures can be initiated by numerous factors including, but not limited to, neurochemical changes and altered receptor dynamics, synaptic morphological changes, genetic and epigenetic factors, neuroinflammatory responses, and extracellular matrix (ECM) changes (36).

**Figure 2:**
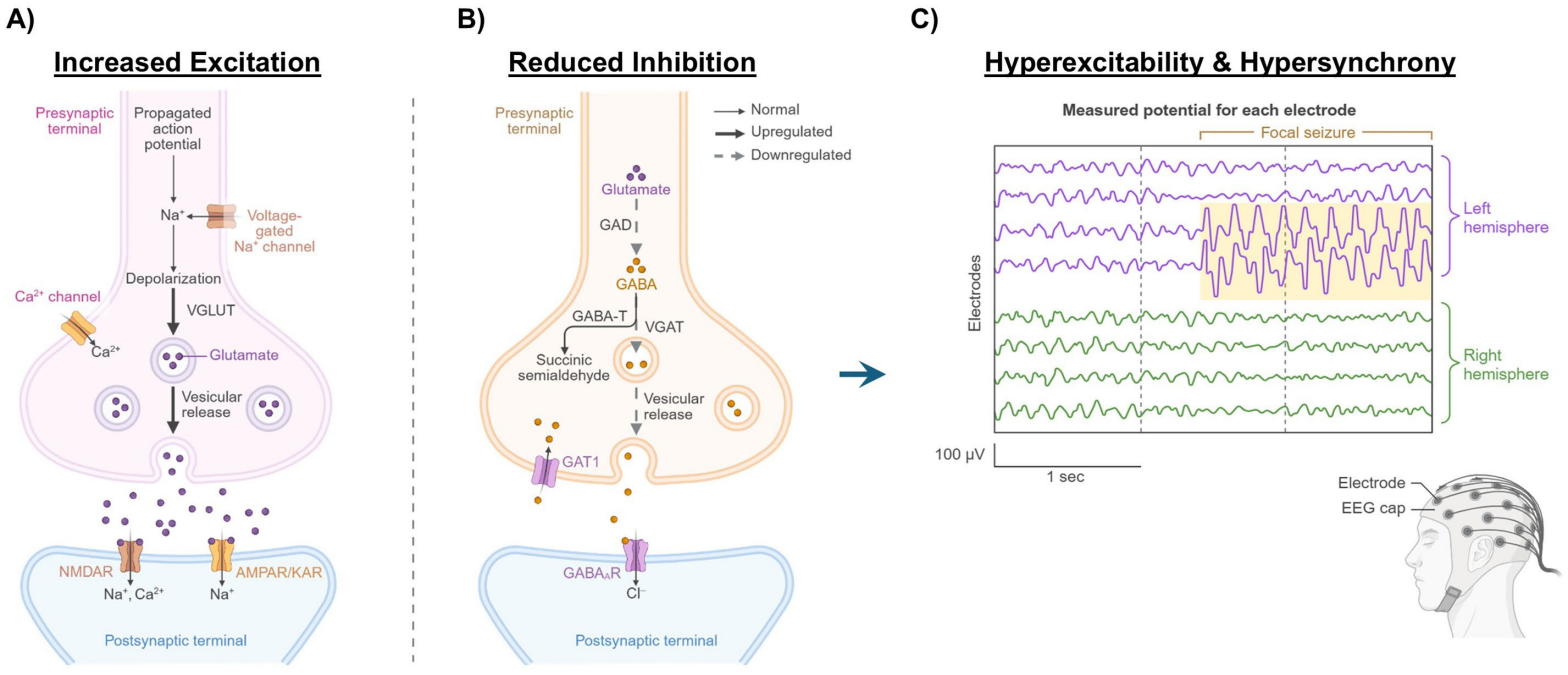
Excitatory/inhibitory (E/I) imbalance. Increased excitation and reduced inhibition supports neuronal network hyperexcitability and hypersynchronization, leading to E/I imbalance and reduced seizure threshold. **(A)** Depolarization of presynaptic glutamatergic neurons increases influx of calcium ion (Ca^2+^) through Ca^2+^ channels within the presynaptic terminals, leading to increased vesicular release of glutamate. Glutamate-mediated activation of N-methyl-D-aspartate receptors (NMDARs) increases postsynaptic Ca^2+^, activating second messengers that increase trafficking of NMDAR and α-amino-3-hydroxy-5-methyl-4-isoxazolepropionic acid receptors (AMPAs) to the postsynaptic terminal, strengthening glutamatergic signaling, and increasing the overall excitability of the neuron. **(B)** Within seconds to minutes of seizure-induced hyperexcitability, dysregulation of neuronal networks through desensitization of gamma-aminobutyric acid (GABAergic) interneurons can occur. Internalization of GABA type A (GABA_A_) receptors on postsynaptic neurons reduces the inhibitory control of the neuronal network. **(C)** The combination of increased excitation and reduced inhibition leads to an E/I imbalance that reduces the seizure threshold, increasing the probability of spontaneous, recurrent seizures. Figure created in BioRender. EEG, electroencephalogram; Cl^−^, chloride; GABA-T, GABA transaminase; GAD, glutamate decarboxylase; GAT1, GABA transporter 1; KAR, kainate receptor; Na^+^, sodium ion; VGAT, vesicular GABA transporter; VGLUT, vesicular glutamate transporter.

E/I imbalances can themselves lead to further molecular changes that further increase excitatory drive and/or reduce inhibitory drive. For example, changes at the cellular level that increase Na^+^ channel activity, glutamatergic signaling, or network connectivity can enhance overall excitatory drive; whereas reduction in K^+^ channel or GABAergic activity lowers overall inhibition and can create E/I imbalance that may increase seizure activity (Figure 2). These molecular changes can increase seizure susceptibility, duration, and severity.

The majority of seizures are self-limiting, resolving in a matter of seconds to minutes; however, seizures can evolve over the course of minutes to become self-sustaining, increasing the risk of treatment intractability, predisposition to spontaneous seizures, and duration of both the current and subsequent seizure(s) (37, 38). As pathological neuronal circuits strengthen, the chance of future seizure occurrence increases, and vice versa, which has been modeled using *in vitro* neuronal cultures (39). Animal models support the idea that hyperexcitability-related dysregulation of neuronal ensembles can occur rapidly and may precede seizure onset, with a loss of synaptic inhibition through desensitization and alterations in kinetic properties of the *γ*-aminobutyric acid type A (GABA_A_) receptors (37, 38). Internalization of synaptic GABA_A_ receptors during sustained hyperactivity leads to a reduction in receptor availability and contributes to loss of benzodiazepine sensitivity and therapeutic response (37, 38). The rapid trafficking of synaptic NMDA receptors to the cell surface enhances synaptic excitability by increasing postsynaptic Ca^2+^ and subsequent activation of CaMKII (37, 38). The increased activation of CaMKII leads to further downregulation of GABAergic signaling and increases AMPA receptor trafficking to the synapse and AMPA receptor conductance (37–40). In addition, NMDA receptor–mediated increases in Ca^2+^ and subsequent activation of protein phosphatase 1 (PPI) leads to the dephosphorylation of KCC2, thus eliminating the GABAergic hyperpolarization mediated by KCC2 (41). Positron emission tomography studies have demonstrated that patients with focal epilepsy have significant increases in surface expression of NMDA receptors, which could contribute to synergistic increases in excitatory drive and reduction in synaptic inhibition (42) (Figure 2). Receptor dynamics leading to E/I imbalance are complex, yet we can evaluate the change in hyperexcitability using MRI techniques to calculate the Hurst exponent (43). Patients with TLE demonstrate a reduced Hurst exponent for the whole brain and significant clusters, indicating a significant increase in E/I imbalance (43). Additionally, excitatory neuronal firing rates are increased in resected tissue from patients with DRE versus tissue from patients without epilepsy (44). Taken together, these cellular mechanisms aid in creating an E/I imbalance that supports self-sustaining seizure activity (37).

### 4.2 The seizure scar develops

The memory of a seizure can be conceptualized as a functional and microstructural “scar” that undergoes structural remodeling to stabilize hyperexcitable circuits and lower the threshold for future events. The brain remembers seizure activity through mechanisms similar to those that drive LTP and LTD. The seizure ensemble’s hyperexcitable and hypersynchronous state leads to a rapid increase in dendritic spine number (45, 46). When a circuit is overwhelmed with hyperexcitability, as seen during a seizure, there is a subsequent increase in dendritic spines, and these spines form mature synapses, as evidenced by an increase in spine density and a concurrent increase and colocation of presynaptic (vGLUT-1) and postsynaptic (PSD-95) elements (45).

As seizure activity continues, additional morphological changes occur in the ultrastructure of affected neurons (45–47). In addition to the increased number of mature synapses, seizures leave a scar across the neuronal ensemble in the form of structural changes that functionally lead to sustained enhancement of synaptic gain and hyperexcitability (45, 48). Several additional structural changes contribute to this enhanced synaptic gain. When neurons continue to experience repetitive glutamatergic stimulation, they need to adapt to the overstimulation; significant changes in the dendritic arbors are required to be able to receive and efficiently process the inputs. Hyperexcitability leads to bifurcation of postsynaptic densities (i.e., perforated synapses), adding synapses to the seizure ensemble (45, 48). Seizure activity also increases the number of spinules that grow from spines, forming even more synapses within the maladaptive circuit (45). The remodeling of individual synapses, neurons and neuronal networks leads to an increase in overall synaptic gain in human pathology (44, 49). Resected tissue from patients with progressive focal seizures and focal-to-bilateral tonicclonic seizures have shown increased mossy fiber sprouting, asymmetric synapse formation, and a greater number of perforated synapses (44, 49). All of these changes lead to enhanced synaptic gain and significantly reduce the seizure threshold, thus promoting a more hyperexcitable neuronal ensemble (45, 48).

### 4.3 The seizure scar deepens: reinforcing circuitry hyperexcitability and hypersynchronization

Activity-dependent myelination plays a critical role in fine-tuning neuronal network dynamics (50, 51). Within hours of the neuronal activity associated with learning and memory or experiential stimuli, oligodendrocyte progenitor cells (OPCs) proliferate and differentiate to mature oligodendrocytes, and axonal myelination of activated neuronal networks increases (52). Evidence suggests that myelin dysregulation plays a role in epilepsy, with an overall reduction in myelin content with progressive disease (53–57). Imaging studies have demonstrated that in later stages of disease, significant gliosis, oligodendrocyte loss, myelin reduction, and myelin dysregulation occur in patients with epilepsy (53–57). However, these studies typically evaluated patients who have progressed significantly in their disease, and few studies evaluated the role of myelination in epileptogenesis and patients with less severe epilepsies or earlier stages of disease (58, 59). Growing evidence in both animal models and clinical studies suggests that in certain epilepsies and certain brain regions, maladaptive myelin plasticity and enhanced myelination may increase seizure susceptibility and progression (50–52, 60, 61). Notably, there are technical limitations when conducting myelin mapping studies, as well as potential differences in brain region or cortical depths that may go unresolved (61, 62). Recent research suggests that myelin reduction is not the only mechanism that leads to network dysregulation in epilepsy, and based on the temporal nature of the disease and the progression of seizures, hypermyelination may also contribute to reinforcing the seizure circuitry, leading to disease progression (50, 63).

The aberrant pattern of neuronal activity in seizures is required for the maladaptive myelination that occurs after epilepsy onset (51). Moreover, the changes in OPC differentiation and myelination are specific to the neurons within the seizure neuronal ensemble (64).

Normal neuronal function also depends on neuron-to-OPC and GABAergic interneuron-to-OPC synapses (50). Aberrant seizure activity increases glutamate release, activating AMPA receptors on OPCs and increasing oligodendrocyte survival and myelination of neurons within the seizure network (51, 64). Increased myelination throughout the seizure neuronal ensemble has been shown to be AMPA receptor and BDNF–TrKB dependent (51). The maladaptive hypermyelination that occurs with seizure activity reinforces the rapid transmission of aberrant neuronal activity, leading to continued network dysfunction and increasing seizure susceptibility (50). This maladaptive myelination is able to temporally and spatially integrate synaptic inputs and synchronize activity across the seizure network (50, 52). This establishes a positive feedback loop where the seizure activity stimulates myelination throughout the network, and the aberrant myelination increases the conductance velocity through impacted neuronal axons that further promotes hypersynchronicity (50, 52).

Due to its impact on the thalamocortical circuitry, maladaptive myelination is also implicated in generalized seizures and seizure progression (50). Thalamic nuclei are involved in disease progression in both generalized–onset seizures and focal-to-bilateral tonic–clonic seizures (65). Neuronal projections from the corpus callosum connect cortical neurons across the hemispheres, which allows for seizures to propagate throughout the brain via myelinated tracts that interconnect the thalamus and cortex (51, 52, 65). In rodent models of generalized seizures, increases in oligodendrogenesis and pathological myelination are present in temporal and anatomical patterns that parallel seizure activity (51). Seizure-induced myelination contributes to network hypersynchrony across the thalamocortical circuitry, which is dependent on BDNF-mediated activation of TrkB, linking the contralateral hemisphere and leading to seizure progression (51, 52). Advanced neuroimaging studies, including diffusion MRI and connectomic analyses, provide evidence for early white matter alterations in epilepsy (66), as well as structural reorganization following surgery and seizure control (67). These findings support the hypothesis that remodeling at the level of structural connections may play a role in seizure propagation. Changes in myelination are dynamic in epilepsy and vary by syndrome, duration, and affected regions. Nevertheless, spatial–temporal mapping alone may miss important disease-related nuances (61, 68–70). Evidence suggests that axonal loss within seizure circuits may follow demyelination or abnormal myelination (70). In high-field MRI and histology studies of TLE, Garbelli et al. found that patients with gray–white matter blurring (a possible indicator of disease progression in the absence of dysplasia) had earlier onset, longer duration, and reduced fractional anisotropy compared with those without blurring, reflecting greater axonal loss or injury (4, 61, 70, 71). Although myelin thickness did not differ, blurring was associated with nonhomogeneous myelin staining and markedly reduced axon number and density (70). These findings suggest that demyelination may precede axonal damage and that increased myelination of select fiber pathways may contribute to seizure-related hyperexcitability.

Quantitative T1 mapping revealed that compared with healthy controls, patients with TLE had increased intracortical myelination, especially in upper cortical layers, independent of cortical thinning (62). These changes were more pronounced in early-onset epilepsy and linked to reduced connectivity with prefrontal networks, suggesting widespread network-specific hyperexcitability (62).

Taken together, these findings indicate that myelin changes may be progressive over time, with increased myelin plasticity leading to seizure susceptibility and progression, gliosis, myelin content reductions, and disease progression leading to dysregulation and continued seizure vulnerability.

### 4.4 The injury emerges

The complement system acts as a sensor of tissue distress (72, 73). In the central nervous system (CNS), the complement system proliferates to protect the brain from neuronal cell injury, damage, and/or death (72). Moreover, neuroinflammatory proteins can function as neuromodulators and can directly impact neuronal function, plasticity, and excitability (72, 73).

Elevations in neuroinflammatory proteins can begin during the seizure and persist for days to months after a seizure event, which may explain the degree of injury over time and increased seizure susceptibility (73–75). Hence, inflammatory proteins have an expanded role in the CNS; in addition to responding to injury, these proteins maintain normal brain function (76–82). Proinflammatory cytokines and proteins associated with the innate and adaptive immune systems contribute to brain development, synapse formation, activity-dependent synapse morphological changes, neural stem cell regulation, and adult neurogenesis, regulating synaptic transmission and plasticity, as well as homeostatic plasticity (76–82).

**Figure 3:**
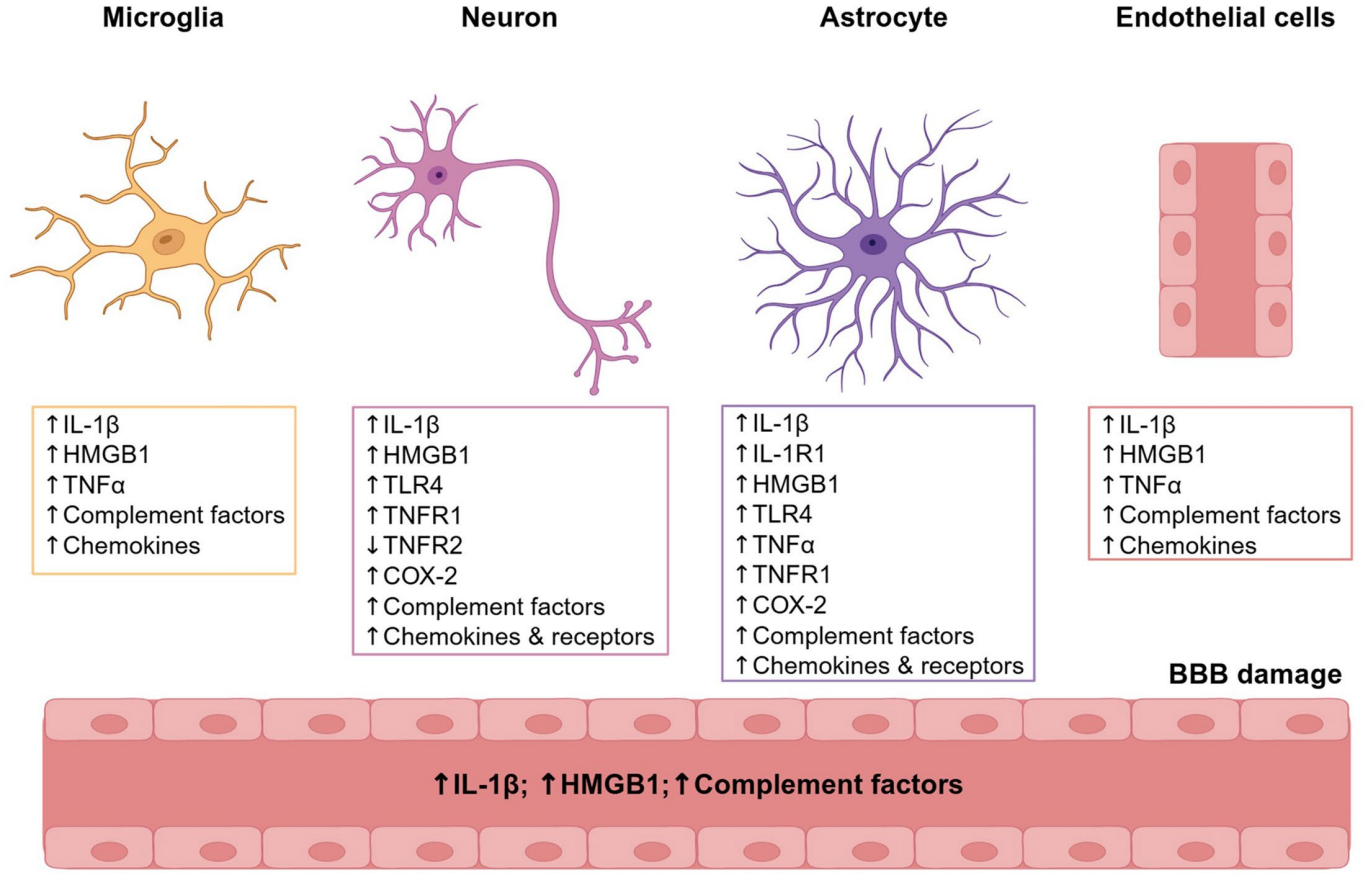
Neuroinflammatory mediators of epilepsy. Many different cell types are involved in the neuroinflammatory response initiated by seizure activity, including microglia, neurons, astrocytes, and endothelial cells. Changes in the expression of certain neuroinflammatory proteins can occur within minutes to hours after a seizure event and can lead to the progression of epilepsy by increasing excitatory drive and loss of inhibitory control neuronal networks. Neuroinflammatory mediators of epilepsy are listed in the figure and reviewed in van Vliet et al. (73). Figure created in BioRender. BBB, blood–brain barrier; COX-2, cyclooxygenase-2; HMGB1, high-mobility group box 1 protein; IL-1β, interleukin-1-beta; IL-1R1, interleukin-1 receptor type 1; TLR4, toll-like receptor 4; TNFα, tumor necrosis factor alpha; TNFR1, tumor necrosis factor receptor 1; TNFR2, tumor necrosis factor receptor 2.

As reviewed in Figure 3, numerous neuroinflammatory modulators are upregulated during or after a seizure (73). Specific neuroinflammatory pathways that are upregulated during seizures in several cell types (i.e., neurons, microglia, astrocytes, and endothelial cells, blood–brain barrier) can affect synaptic function and plasticity via direct effects on receptor systems and ion channels (73). Here we focus on several neuroinflammatory molecules that increase the risk of seizure progression, frequency, and duration, which have been shown to increase with severity of disease in patients with epilepsy (83).

TNFα is a proinflammatory cytokine known for its role in the innate immune response (84). During injury or infection, TNFα expression is increased, and contributes to inflammation, apoptosis, and cell death (84).

In the CNS, TNFα mediates normal synaptic plasticity and synaptic responses due to injury (79, 80). TNFα released by astrocytes activates TNF receptors on postsynaptic neurons that increase AMPA receptor trafficking, leading to increased spontaneous mini excitatory synaptic currents (80). In addition, TNFα is required for maintaining the surface expression of AMPA receptors and synaptic strength (80).

During seizure, there is a rapid induction of TNFα both in glial cells and within the endothelium of the blood–brain barrier, and TNFα expression can remain elevated for up to 3 days following seizure (73, 85–88). Along with the increased surface expression of AMPA receptors, TNFα reduces GABA_A_ receptor surface expression through endocytosis (79). Overall, TNFα can shift the E/I balance in neurons through changes in glutamatergic and GABAergic receptor trafficking, which strengthens the excitatory synapse (79, 80, 89). If maladaptive, this function could lead to overexcitation and subsequently cause neuronal damage or cell death (79). In addition, the presence of TNFα during seizure onset increases seizure susceptibility and frequency (87).

The interaction between TNFα and both the glutamatergic and GABAergic receptor systems may contribute to reinforcing and maintaining seizure susceptibility and progression by the activity-dependent increase in AMPA receptor surface expression and GABA_A_ receptor internalization, thus priming neurons for hyperexcitability (73, 79, 87).

Interleukin-1 beta (IL-1β) is a proinflammatory cytokine known for its role in the innate immune response to injury and/or infection (73, 82, 90, 91). Although IL-1β plays a role in CNS inflammation, and its expression during chronic disease can exacerbate damage, it also modulates neuronal function (73, 82, 91, 92).

IL-1β expression is induced during seizure and seizure recurrence and may be a biomarker for epileptogenesis, epileptic foci, and seizure progression (73, 92). In a healthy brain, IL-1β mRNA and protein expression is barely detectable; however, its expression is rapidly upregulated within ≤30 min of seizure activity (73, 85, 92–95). Depending on seizure severity, increased IL-1β expression may be transient during the acute phase of the seizure and returns to normal levels over 48 to 72 h (95). Interestingly, in a model of febrile seizure, rodents that experienced febrile seizure–induced status epilepticus were at a higher risk for developing spontaneous seizure, which is associated with a persistent increase in IL-1β (95). Furthermore, patients with DRE due to focal cortical dysplasia or glioneuronal tumors had elevated levels of IL-1β and IL-1 receptor type 1 (IL-1R1) in multiple cell types, including neurons and glial cells (94). Differential IL-1β and IL-1R1 upregulation in aberrant neurons can contribute to increased excitability in the seizure neuronal network, leading to seizure progression (85, 94–96).

The IL-1β/IL-1R1 pathway also enhances neuronal hyperexcitability by increasing AMPA receptor expression, glutamate release, and NMDA receptor–mediated Ca^2+^ influx, leading to excitotoxicity and cell death. Elevated IL-1β also drives astroglial activation and prolonged glutamate elevation, while reducing GABAergic inhibition. These effects are evident in patients with TLE and can be counteracted by increasing IL-1Ra or administering intravenous immunoglobulin, which inhibit IL-1β/IL-1R1 signaling, highlighting the role of this pathway in seizure progression (97–99).

High-mobility group box protein 1 (HMGB1) is a ubiquitous inflammatory protein found in nearly all cell types in the body (81, 100). HMGB1 is present in the nucleus, where it acts as a transcription regulator, and also in the cytoplasm, where it can act as second messenger; it also has a role in extracellular activation of toll-like receptor 4 (TLR4) (73, 81, 100). As HMGB1 activity is ubiquitous, administration of the protein leads to systemic inflammatory responses, and HMGB1 antagonists can reduce the severity of inflammatory disease and lethality in animal models (81). HMGB1-mediated activation of TLR4 is the primary pathway supporting macrophage activation, cytokine release, and tissue damage, which is associated with the immune response (73, 81, 100).

Patients with DRE have increased activation of HMGB1/TLR4 signaling (73, 101–103). During seizure, the HMGB1/TLR4 pathway is activated, leading to downstream phosphorylation of NMDA receptors, increased intracellular Ca^2+^, and reinforced hyperexcitable neuronal states (73, 97, 103). In addition, HMGB1 is associated with reduced GABAergic signaling, increased glutamate release, and inhibited glutamate reuptake, which increases extracellular glutamate and contributes to E/I imbalance that reinforces hyperexcitability (73, 97, 103, 104). In animal models, antagonism of the HMGB1/TLR4 pathway can reduce seizure frequency and seizure duration, further implicating this pathway in seizure progression (103, 105). It has been suggested that HMGB1 may be a biomarker for both epileptogenic and seizure progression (103).

Cyclooxygenase-2 (COX-2) is an enzyme that synthesizes prostaglandins involved in response to inflammation and other physiological stimuli to mediate pain and inflammatory processes (73, 106, 107). Following brain injury and during seizure, increased COX-2 expression in neurons leads to the production of prostaglandins, including prostaglandin E2 (PGE2) (73, 74, 107, 108). Elevations in COX-2 can last for hours to days following prolonged seizure, status epilepticus, and repetitive seizures (73, 107, 109, 110). Interestingly, COX-2 expression is increased during the acute phase of seizure, returning to baseline levels during the latent phase and increasing once again during the chronic phase, with the presentation of spontaneous recurrent seizures (73, 93, 108, 109, 111).

COX-2 expression is normally upregulated by increased neuronal activity. During a seizure, rapid upregulation of COX-2 results from increased NMDA receptor–dependent seizure activity (73, 109, 112). COX-2 upregulation leads to the production of PGE2, which binds to the prostaglandin E (EP) family of receptors, enhancing the neuroinflammatory response, neuronal injury, and presynaptic glutamate release, thus supporting the hyperexcitability of postsynaptic neurons (107–110).

COX-2 contributes to epilepsy comorbidities, including sudden unexpected death in epilepsy (SUDEP), by regulating sleep–wake cycles and promoting vasoconstriction through prostaglandin-mediated activation of L-type calcium channels, leading to postictal hypoperfusion and hypoxia (110, 113). Its expression also enhances fever responses, linking it to febrile seizures (110, 114, 115). Beyond seizure activity, COX-2 has been associated with depression, anxiety, and cognitive deficits, suggesting a broader role in long-term consequences of DRE (108, 110).

### 4.5 Treatments implicating the neuroinflammatory system in epilepsy progression

Several preclinical and clinical studies showed that targeting neuroinflammation can reduce seizure burden (73, 74, 107). Aspirin may decrease seizures in focal epilepsy and Sturge–Weber syndrome, whereas ibuprofen shows no benefit in febrile seizures (116–118). Modulation of cytokine pathways, such as IL-1β blockade with anakinra or ICE inhibitors and TNFα inhibition with adalimumab, reduced seizure frequency in febrile infection–related epilepsy syndrome (FIRES), refractory status epilepticus, and Rasmussen’s encephalitis (119–123). Overall, these findings implicate neuroinflammation in seizure development and progression (123).

### 4.6 Remodeling of the ECM

The ECM provides critical structural support for neurons and contributes to overall brain function: it is involved in neuronal development, cell migration, neurite outgrowth and neuronal morphology, signal transduction, and synaptic plasticity (124–130). As a dynamic lattice, the ECM regulates the microenvironment around cells, playing roles in both normal brain activity and pathophysiology (124–126, 130–133). The major structural components of the ECM found in the brain include perineuronal nets (PNNs), basement membranes (BMs), and axonal coats (124, 125). PNNs play a critical role regulating GABAergic inhibitory signaling, which in turn regulates glutamatergic firing (124, 128, 129, 131). Under normal physiological conditions, PNNs can decrease the membrane capacitance of GABAergic interneurons, increasing both the release of GABA and inhibitory control of glutamatergic neurons (131). PNNs lose control over GABAergic interneurons, as increased expression and activity of matrix metalloproteinases (MMPs), a disintegrin and metalloproteinase with thrombospondin motifs (ADAMTs), and tissue inhibitors of metalloproteinases (TIMPs) lead to PNN destruction, thus increasing GABAergic interneuron membrane capacitance and prolonging reduced GABA release (131). This reduced inhibitory tone increases excitatory drive and hyperexcitability of glutamatergic neurons, promoting more seizures (131–134).

In addition to its effects on the GABAergic system, the ECM directly impacts the glutamatergic system, increasing hyperexcitable states (125, 128, 129, 131, 134–138). Components of the ECM alter the localization of AMPA and NMDA receptors in dendritic spines and on dendritic shafts (128, 136). The ECM can also alter AMPA receptor properties, increasing burst firing duration, decreasing interburst intervals, and prolonging the open state of these channels by 3 to 5 fold, thus promoting hyperexcitability (128, 137, 138).

Several developmental epilepsies are characterized by malformations and associated with overexpression of ECM proteins (e.g., tuberous sclerosis complex, gangliogliomas, and focal cortical dysplasia) (126). In addition, patients with intractable mesial TLE (MTLE) show changes in ECM protein expression and reductions in PNNs, which positively correlate with seizure episodes (133).

When evaluating the clinical progression of MTLE and considering age at epilepsy onset, duration of epilepsy, number of ASMs, and frequency of seizures, patients with lower seizure frequency showed less diffuse staining of ECM proteins (133). To maintain functional connectivity (FC) and appropriately modulate excitability, specific patterns of ECM protein expression are both condensed and diffuse (133). In patients with MTLE, both condensed and diffuse ECM structural organizations are disrupted (133). This changes the composition and distribution of PNNs, which reinforces E/I imbalance and seizure progression (131–134). The continuation of spontaneous seizures over time reinforces the maladaptive reorganization of the ECM and breakdown of PNNs, supporting progression of disease (133).

The ECM has been targeted as a potential treatment option for certain epilepsies due to its contribution to E/I imbalance in seizure circuitry (134, 139, 140). It is reasonable to think that restoring ECM volume, reorganizing PNNs, and stabilizing GABAergic interneuron function may help reduce seizure burden (134).

## 5 Reinforcing the continuation of seizure and treatment intractability

### 5.1 Genetic and epigenetic factors underlying seizure and epilepsy

Additional factors contribute to the phenotypic expression of seizure and epilepsy (141–144). Genetic mutations can precipitate or initiate neurochemical, structural, functional, or circuitry changes that lead to seizure progression by reducing seizure threshold by creating an E/I imbalance, which promotes hyperexcitability and hypersynchronicity. To date, more than 1,000 genes have been associated with epilepsy, which are reviewed elsewhere (https://github.com/bahlolab/genes4epilepsy) (141, 144).

The conversion of experience into genetic modifications that influence cellular events, or epigenetics, plays an important role in the initial presentation and progression of seizure and has a long-term, cumulative impact on the patient (145–147). Several common processes define epigenetics, including DNA methylation, histone acetylation, and non-coding RNA (ncRNA) expression changes, which have all been associated with the development and progression of seizures (148–150).

Although the methylation of promoter regions of genes is typically associated with gene silencing, considered very stable, and can prevent transcription, DNA methylation at alternative sites within the sequence is associated with increased transcription, demonstrating that DNA methylation is a dynamic process that modulates gene expression (149, 151, 152). Furthermore, DNA methylation is modulated by neuronal activity (149, 153). Experience-dependent, neuronal activity–induced DNA methylation changes in the mature brain can be dependent on NMDA signaling (153). Additionally, the changes in methylation status are long-acting, suggesting that activity-induced changes in methylation may influence epileptogenesis and seizure progression (149, 150, 153).

Changes in DNA methylation patterns have been identified in patients with epilepsy (149, 154–156). The link between an initial seizure and long-lasting changes in DNA methylation patterns that promote further or spontaneous seizure activity has been investigated (157). AMPA receptors that include heterodimer structures integrating GluA2 subunits are impermeable to Ca^2+^, whereas Glu1A subunits are inwardly rectifying and promote Ca^2+^ permeability (157–159). Studies have demonstrated that rodents exposed to kainic acid to induce epileptiform activity have a long-lasting increase in DNA methylation, leading to silencing of GRIA2 and a subsequent reduction in *gria2* mRNA expression, thus increasing AMPA receptor Ca^2+^ permeability and neuronal burst firing and promoting spontaneous seizure activity (157). Changes in DNA methylation patterns due to seizure activity have been seen with other genes that contribute to E/I imbalance, including *GRIN2B* (NMDA receptor subunit) and *BDNF*, and contribute to the progression of seizure activity (160). This is translatable to humans, as there is a shift in AMPA receptor subunit expression in patients with epilepsy, including downregulation of GluA2 and upregulation of AMPA receptor subunits that promote Ca^2+^ permeability and increase susceptibility to epileptiform activity (161).

Similar to DNA methylation, posttranslational modification (PTM) of histones (i.e., acetylation, phosphorylation, methylation, ubiquitylation, and sumoylation) influences gene transcription, leading to increased or decreased gene expression (149, 150, 162, 163). Histones are proteins that organize DNA and provide structural support to chromosomes (146, 147, 164, 165). Histone PTMs play critical roles in gene transcription, replication, repair, and changes in genetic architecture through chromatin remodeling (164, 165).

Both acute and chronic seizures have been shown to impact histone PTM (145). Histone acetylation alters gene transcription following neuronal activity: increases in histone acetylation can be observed 30 min following an acute seizure event and can last hours before returning to baseline levels (145). In the chronic seizure state, a seizure can initially increase histone acetylation, followed by a marked reduction 24 h post-seizure, indicating the longer-term structural changes and chromatin remodeling that occur with chronic seizures (145). Specific to neuronal hyperexcitability, following a seizure, the hyperacetylation of *BDNF* leads to increased expression, whereas histone deacetylation of *GluA2* precedes a reduction in the expression of the AMPA receptor subunit (145–147). Seizure-induced upregulation of histone deacetylases (HDACs) could impact the expression of other genes critical to hyperexcitability or the development of comorbidities associated with epilepsy (147, 166, 167). Interestingly, with repetitive electroconvulsive seizure, upregulation of HDAC2 in the frontal cortex led to a reduction in acetylation and expression of the NR2A and NR2B subunits of NMDA receptors, along with a reduction in CaMKIIα expression (166, 167). This is a critical finding, as it could provide evidence for dysregulation of impacted neuronal circuitry and provide a basis for the learning and memory deficits experienced by patients with epilepsy (151, 168). Notably, patients with TLE have increased HDAC2 expression, which is proposed to disrupt synaptic plasticity through dysregulation of gene expression and long-term chromatin remodeling (167).

ncRNA has also been implicated in epilepsy (149, 150). ncRNA, consisting of microRNAs, long ncRNAs (lncRNAs), and circular RNAs (circRNAs), have been shown to play important roles in regulating gene transcription and neuronal function, including development of neuronal circuitry and plasticity (169, 170). Soutschek and Schratt reviewed the role of ncRNA in neuronal function and neuronal circuit remodeling (170). The development and stability of neuronal function has been shown through loss-of-function experiments in which loss of function of certain microRNAs leads to reduced miniature excitatory postsynaptic currents (mEPSCs), pointing to a reduction in overall excitatory synapses (170). Loss of other microRNAs impacted GABAergic system maturation, leading to structural changes in neurons that increase spontaneous, synchronous excitatory activity (170). Additionally, microRNAs can impact genes required for synaptic vesicle release and AMPA receptor trafficking, demonstrating their role in regulating excitatory synaptic strength (170). Overall, ncRNAs are critical in the development and maintenance of neuronal integrity and regulate the expression of multiple genes involved in synaptic transmission, synaptic plasticity, and long-term homeostatic plasticity maintenance (170).

The involvement of ncRNAs in neuronal structure and function suggests that ncRNAs could play a role in epileptogenesis, as well as long-term seizure progression. Notably, 2 months following a seizure, levels of over 100 microRNAs either increased or decreased in patients with epilepsy (171). The expression of several core ncRNAs are consistently altered with seizure and epilepsy, including miR-34a, miR-132, and miR-188, which are associated with neuronal death following status epilepticus (149, 150, 171).

Increased levels of miR-134 reduces seizure threshold through modulation of dendritic spine proteins (149, 171, 172). In addition, antagonism of miR-134 reduces the development of epilepsy or spontaneous seizure following status epilepticus (149, 171, 172). Moreover, in animal models, pretreatment with miR-134 antagonists prior to initiation of status epilepticus reduced seizure duration, leading to reduced neuronal damage (172). Loss of miRNA-128 increased overall spine density, enhanced neuronal excitability, and led to fatal epilepsy in rodent models, whereas ectopic expression of miR-128 in postsynaptic, excitatory neurons reduced seizure frequency (149, 173). lncRNAs can also influence the development and progression of seizures (149). For instance, increased expression of metastasis-associated lung adenocarcinoma transcript 1 (*Malat1*) increased mature synapse formation, promoting hyperexcitability (174). Reduced seizure threshold and increased seizure frequency are associated with reduced brain cytoplasmic 1 (*BC1*) RNA (175). *BC1* RNA is critical for neuronal excitatory suppression, and loss of *BC1* RNA leads to increased prolonged spontaneous burst firing of glutamatergic neurons, increasing hyperexcitability and continued propensity for prolonged spontaneous burst firing (175). Taken together, these data suggest that alterations in ncRNA expression can have structural and functional impacts on neuronal circuitry that lead to a reduction in seizure threshold and continuation of seizures. Additionally, following an initial insult leading to seizure, ncRNAs may be useful to suppress the underlying epileptogenesis processes during the latent phase, reducing or eliminating the development of spontaneous seizure activity (chronic phase). Increasing research is focused on developing ncRNA as biomarkers and treatment for epilepsy (176).

## 6 Long-term consequences of continued seizure and brain health

The brain is organized into large-scale networks whose structural and functional relationships are altered in disease (162). The structural and functional characteristics of human brain networks and their connections can be studied *in vivo* using imaging techniques, namely diffusion tensor tractography and blood oxygen level–dependent (BOLD) signal in functional MRI (fMRI).

Widespread structural and FC alterations are detectable in both generalized and focal epilepsy, beyond the epileptogenic zone and across cortical and subcortical regions (163, 177–182). In patients with TLE, resting-state fMRI revealed an imbalance between local and long-range connections, in which aberrant local connectivity and reductions in long-range projections led to network segregation; the structural organization of white matter architecture is independent of cortical atrophy and reduces seizure threshold (181–183). For example, although there is a reduction in intrahemispheric FC in patients with TLE, local ipsilateral FC is increased, with higher nodal centrality in the thalamus, anterior cingulate cortex, and fusiform gyrus (182).

The functional network can be explained in part by monosynaptic and oligosynaptic structural connections, and changes in this relationship between structural connectivity (SC) and FC, or SC-FC coupling abnormalities, have been described in both focal and generalized epilepsy (162, 178, 181, 184). Moreover, SC-FC coupling abnormalities are thought to worsen with increased epilepsy curation and disease progression (163, 177, 178, 181). Patients with generalized epilepsy have an overall reduction in SC-FC coupling compared with healthy controls, and the strength of the SC-FC coupling negatively correlates with the duration of epilepsy (162). This suggests that SC-FC decoupling may contribute to long-term progression of disease (162). Similar changes in SC-FC coupling have been described in patients with TLE (178, 181, 184). Studies have demonstrated that changes in SC-FC coupling are dynamic and change throughout disease progression (181, 184).

Due to network connections between brain regions, each network “node” can influence other nodes; continued seizures impact local networks within an epileptogenic foci and can cause widespread neuropathologies, depending on epilepsy duration and the affected nodes and brain regions (43, 162, 181, 182, 184–186). In addition to seizures, the main manifestation of the epileptic network, the development of comorbid cognitive impairments and psychiatric illnesses has been of outmost interest. Central to the distributed network are the main default mode network (DMN) “hubs”: the medial prefrontal cortex, posterior cingulate, precuneus, and angular gyrus, as well as other highly connected regions central to distributed networks (e.g., hippocampus, amygdala, striatum, and thalamic nuclei). The DMN is critical to executive functioning, working memory, and attention; DMN dysfunction can lead to cognitive impairment, anxiety, and depression and can contribute to seizure progression, demonstrating that the widespread impact of epilepsy to distal brain networks outside the seizure foci (177, 182, 187). Several neurocognitive deficits have been linked to alterations in FC across the DMN that contribute to attention, language, visuospatial working memory, and executive functioning (162, 177, 179, 185, 186, 188–190).

When assessing SC and FC in patients with epilepsy, possible confounds due to the ASMs being administered should be considered (188, 191, 192). Furthermore, as abnormal functional connections are reinforced through continued seizure activity, a reduction in distal normal functional connections may occur as the connections become less active, leading to additional neuropathology (178). There is growing evidence on the utility of SC and FC as a potential biomarker as a measure for treatment responsiveness (193, 194). Patients whose seizures are not controlled with ASMs have significant differences in FC measures compared with both healthy controls and patients whose seizures are well controlled by ASMs, which could potentially help forecast DRE and allow for earlier changes in treatment strategy and disease management (193, 194).

### 6.1 Cell death and atrophy

In addition to structural and functional changes that occur with ongoing seizures, the continuous increase in seizure frequency and duration can activate programmed cell death pathways, which in turn alters cellular function well before neuronal death occurs (195, 196). Based on imaging studies, neurocognitive testing, and functional deficits, repeated seizures impact brain structure and lead to neuronal loss (196–198). There are several hypotheses on how seizure-induced neuronal cell death can lead to increased hyperexcitability, further increasing susceptibility to recurrent seizures (i.e., the recapitulation of development hypothesis and the neuronal death pathway hypothesis) (195). The recapitulation of development hypothesis suggests that neuronal death triggers the formation of new excitatory synapses by preserved neurons in the area impacted by the seizure; the resulting maladaptive circuitry maintains E/I imbalance via the loss of GABAergic interneurons and an increase in newly formed glutamatergic synapses (195, 199, 200). However, acute brain injury that causes neuronal death does not always lead to the development of epilepsy, and epileptogenesis and seizure progression can occur prior to neuronal death. This has led to the development of the neuronal death pathway hypothesis, which postulates that seizure events can activate neurodegenerative processes that precede neuronal death and contribute to the worsening of seizures and non-seizure symptoms (i.e., progression of epilepsy) (195, 196).

Seizures may initiate apoptosis through glutamatergic mechanisms (196, 197). Excessive glutamate release causes overexcitation of NMDA receptors that increases intracellular Ca^2+^ concentrations, which in turn activates excitotoxicity pathways (196, 197, 201). In addition to NMDA receptor–mediated cell death, TNFα plays a role in seizure-induced apoptosis, as seizure-dependent TNFα-mediated activation of TNFR1 induces antiapoptotic genes in impacted neurons (197). After seizure, the cascade of neurochemical, neuroinflammatory, and genetic events can lead to the continuous change and reinforcement of neuronal structure and cell death (197, 202).

Seizure-associated changes are dynamic rather than static, and the underlying cascade of events triggered by an initial seizure can evolve throughout the latent period to strengthen the E/I imbalance, leading to a chronic increase in spontaneous seizures (4, 203). Following the evolution of epilepsy through the latent period, the spontaneous recurrent seizures increase seizure burden, reinforce the pathological neuronal circuitry, and begin to impact wider neuronal networks, which may contribute to comorbidities (163, 177–182, 203).

## 7 Overall impact on patient health and QoL

**Figure 4:**
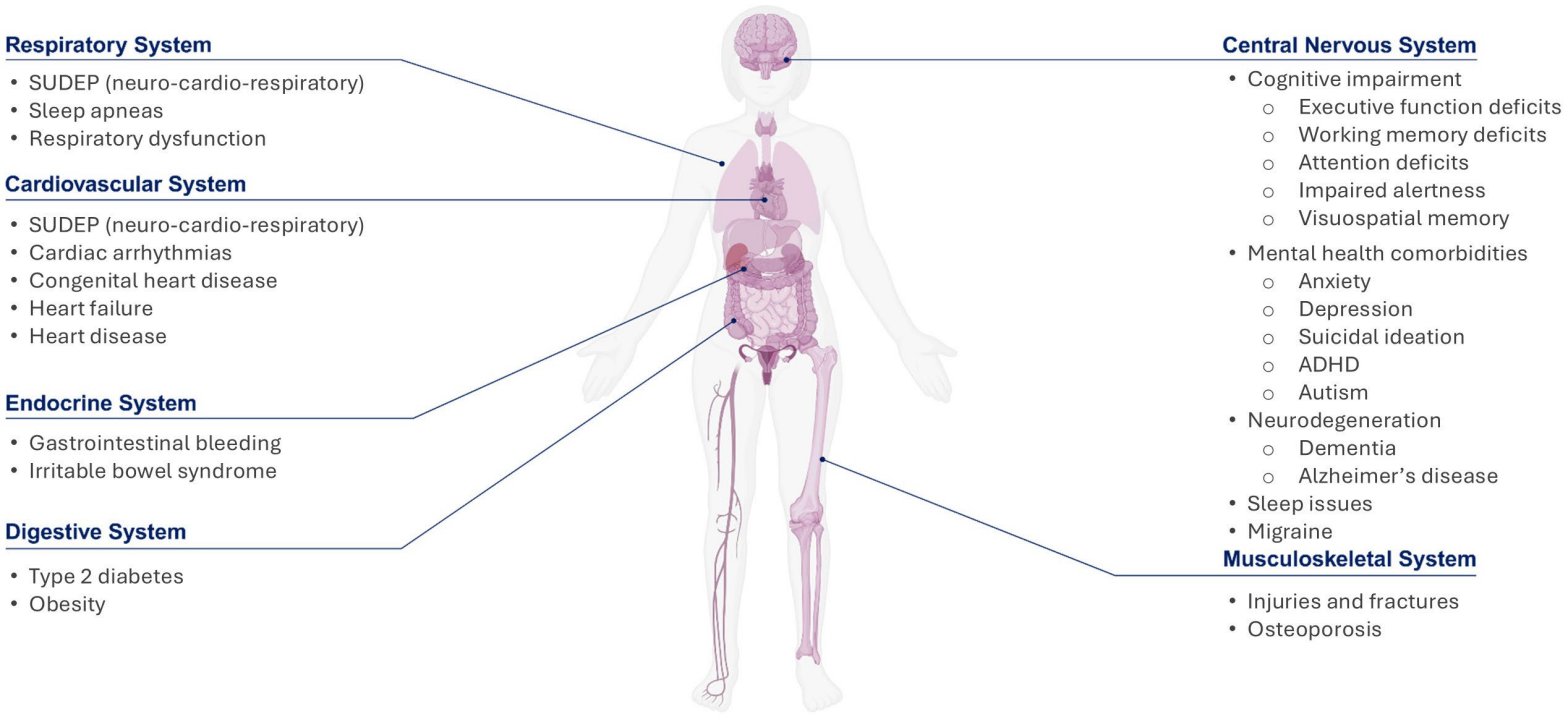
Common comorbidities with epilepsy. Epilepsy is a neuronal network disorder, with pathology often extending past the epileptogenic focus and impacting other areas of the brain that play a role in the central regulation of other systems, which contributes to the development of comorbid conditions. This figure shows common comorbidities associated with epilepsy, highlighting the fact that epilepsy is much more insidious than just seizures, and thoughtful treatment strategies need to be adopted in order to mitigate the progression of seizure and other comorbidities (204–210). Figure created in BioRender. ADHD, attention deficit hyperactivity disorder; SUDEP, sudden unexpected death in epilepsy.

Epilepsy extends beyond recurrent seizures, and treatment should include a multidisciplinary team that does not solely focus on reduction in seizure burden. Comorbidities are common in patients dealing with epilepsy and negatively impact patient QoL (204–210). Common comorbidities experienced by patients with epilepsy are shown in **Figure** **4**. As with any complex disease, the cause-and-effect relationship between epilepsy and specific comorbidities is uncertain as epilepsy or the comorbid condition could cause the other but may also coincide, as they share common underlying mechanisms or mutual causes. Comorbid conditions could result from seizures or from the treatment of seizures (206, 207).

### 7.1 Cognitive impairment associated with epilepsy

Cognitive impairment and decline are common in epilepsy, and the impact of epilepsy on cognition has been well studied (168, 211–216). Cognitive impairments in patients with epilepsy are linked to the underlying disease etiology, and further cognitive decline has been associated with interictal epileptiform activity and recurrent or prolonged seizures (168, 214, 216). Cognitive impairment in patients with epilepsy is in part due to the neurochemical, morphological, genetic, and functional changes discussed above, with frequent prolonged seizures and duration of illness most significantly impacting cognitive function (168, 214).

Epilepsy and ongoing seizures can impact several areas of cognition, including working memory, attention, processing speed, visuospatial memory, language, learning, and long-term memory (168, 177, 181, 214, 215). Many of the over 1,000 genes associated with epilepsy are also associated with cognitive impairment (141, 144, 168, 214, 216). For example, Dravet syndrome (DS) is a developmental epileptic encephalopathy associated with multiple seizure types and neurodevelopmental impairments that lead to cognitive deficits typically beginning in the second year of life (217). The majority of DS cases are caused by a *de novo* mutation in the *SCNA1* gene, leading to decreased Na_v_1.1 voltage-gated Na^+^ channel function (216, 218). The reduced sodium currents in GABAergic interneurons leads to reduced inhibitory activity, leading to further E/I imbalance (217). Although the ongoing seizures experienced by patients with DS can lead to further damage and cognitive issues, the loss of Na_v_1.1 function due to continued seizure contributes to the development and progression of cognitive impairment (219). Communication between the prefrontal cortex and hippocampus is critical for normal cognitive and emotional processing (219). Loss of function of Na_v_1.1 voltage-gated Na^+^ channels leads to altered network activity in the prefrontal cortex in both the active and resting states, which in turn leads to impaired working memory, reduced sociability, and resistance to fear extinction (219). Catterall et al. proposed that severity of illness is based on the extent of Na_v_1.1 function loss, with mild loss of function predisposing patients to familial febrile seizures, further loss of function predisposing patients to generalized epilepsy with febrile seizures, and complete loss of function leading to intractable seizures and more severe comorbidities (217). Further support of this hypothesis was demonstrated in human studies showing that changes in allelic expression of *SCNA1* alter working memory performance and brain activity measured by MRI (220).

Many genetic mutations leading to epilepsies are associated with underlying changes in synaptic plasticity, including other channelopathies that impact voltage-gated K^+^ channels, nicotinic acetylcholine receptors, calcium channels, and GABA receptors; these all contribute to structural and functional changes at the synaptic level that have long-term impacts on cognition and neuronal networks beyond the epileptogenic foci (221–225).

Recurrent epileptic seizures also significantly impact cognition (168, 226). The cognitive effects due to seizure are not static but evolve over time (226). For instance, during an acute seizure event, brief alterations in network excitability and synchronous activity interfere with neuronal coding, memory retrieval, and memory maintenance (226, 227). Moreover, interictal epileptiform discharges (IEDs) can lead to transient cognitive impairment in patients with epilepsy. Depending on the location and timing of IEDs, patients can experience impairments in short-term memory, free recall, short-term verbal memory, and reaction times (227–229). Increased IEDs outside the seizure onset zone have been demonstrated to impact memory encoding in simple recall tasks (228). IEDs can also impact memory retrieval and maintenance, with increased spike activity reducing recall (228, 229).

During the ictal period, spontaneous repetitive seizures lead to persistent changes, which lead to alterations in cellular function that underlie longer-term, evolving cognitive deficiencies in patients with DRE. Holmes reviewed the maladaptive neurophysiological patterns that emerge due to recurrent seizure, which lead to cognitive impairment, namely a reduction in GABAergic inhibitory signaling, impairment in spike frequency adaptation, reductions in the hyperpolarization potentials that follow spike trains, impairments in LTP, alterations in theta power, and alterations in the precision and timing of place cell firing and stability (226). Of course, there are also seizure events (i.e., status epilepticus) that promote acute neuronal death, cell damage, and synaptic changes that negatively impact cognition, which can lead to further spontaneous recurrent seizure and continued cognitive decline (230–232).

The cascade of structural and functional changes that occur with recurrent seizures impact both local and distal neuronal networks, creating altered and maladaptive information processing that impact cognition in patients with DRE. However, ASMs can also contribute to cognitive impairment, and a higher number of ASMs is also associated with worse cognitive outcomes. Nevertheless, disrupted SC and FC due to continued seizure activity is a significant factor contributing to cognitive impairment in patients with epilepsy (168, 177, 181, 213, 214, 216, 226). Regardless of the etiology of cognitive impairment, improved seizure control will benefit cognition over time (214, 216, 226).

### 7.2 The impact of epilepsy on mental health

Patients with epilepsy also commonly experience mental health comorbidities, such as depression and anxiety (205–207, 233). Notably, neuropsychiatric symptoms and lower scores on neuropsychiatric assessments are predictors of future pharmacoresistance, and patients with DRE have higher rates of anxiety and depression compared with those whose epilepsy is controlled without seizure (233–235). An estimated 20 to 30% of patients with epilepsy have a psychiatric comorbidity, with approximately 51 to 70% of patients experiencing a psychiatric disorder in their lifetime (205, 236). The most common psychiatric comorbidities are mood disorder and anxiety (35 and 26%, respectively), and the most common mood disorder is major depressive disorder (MDD; 24%, depending on the population evaluated) (205, 215, 236).

Mood disorder and anxiety are reciprocal comorbidities, meaning that having either epilepsy or these psychiatric disorders increases the risk of developing the other (205–207, 233). The neuronal networks and brain regions impacted by epilepsy often overlap with those implicated in mood disorders and anxiety (206, 233). Whether directly impacted by the seizure within the epileptogenic foci or widespread network dysregulation, disruption within neuronal circuitry in the frontal lobe, temporal lobe, or limbic region can lead to comorbid mood or anxiety disorders (206, 233, 237–242).

Epilepsy and depression share common mechanisms that involve the dysregulation of specific receptor systems, including decreases in serotonergic, noradrenergic, dopaminergic, and GABAergic activity (243). Several imaging studies aimed to elucidate the influence of the serotonergic system, specifically serotonin-1A (5-HT_1A_) receptor activity, on the progression of epilepsy and depression (244–246). A recent meta-analysis examined changes in 5-HT_1A_ binding in patients with TLE (246). This analysis confirmed 5-HT_1A_ receptor binding is reduced in the hippocampus, temporal cortex, amygdala, and frontal lobe ipsilateral to the seizure foci (246). Patients with comorbid TLE and MDD had a further reduction in 5-HT_1A_ binding in the anterior cingulate cortex, hippocampus, and medial and superior temporal lobe, which extended to limbic areas outside the seizure focus (244).

Reduction in 5-HT_1A_ receptor activity is not only associated with epilepsy and MDD but may also reduce seizure threshold, promoting hyperexcitation (247). Activation of 5-HT_1A_ receptors reduces glutamatergic signaling by blocking NMDA receptor trafficking via a CaMKII- and ERK-dependent mechanism that destabilizes microtubules (248).

Thus, by reducing 5-HT_1A_ receptor activity, glutamatergic receptor trafficking is disrupted, increasing the surface expression of NMDA receptors and reducing the seizure threshold (247). Although this is one example of how the serotonergic system can influence excitatory states, the family of serotonergic receptors has become an attractive target for the development of ASMs that may have additional therapeutic effects on neuropsychiatric and cognitive comorbidities (249). Sourbron and Lagae (249) reviewed the therapeutic potential of serotonergic receptors in the treatment of epilepsy.

As with depression, anxiety and epilepsy share common biological mechanisms and are thought to be reciprocal comorbidities involving the amygdala, hippocampus, and hypothalamic–pituitary–adrenal (HPA) axis, among other brain networks (233, 241, 242, 250). Depending on the patient, epilepsy type, and seizure focus, anxiety can have preictal, ictal, postictal, or interictal presentation or could be a long-term comorbidity associated with a common underlying etiology (250). In epilepsy, the reduction in GABAergic inhibition increases excitatory discharge that activates fear and anxiety circuitry within the amygdala, causing E/I imbalance (233, 250–252). Due to reciprocal connections throughout the brain, alterations in brain regions outside of the amygdala can influence the development of different forms of anxiety. Neuronal connections between the amygdala and the medial prefrontal cortex and hippocampus can drive different forms of anxiety including generalized anxiety, fear-based anxiety, novelty-based fear, and social anxiety (240). As with epilepsy, anxiety is a dysfunction of neuronal networks that span multiple brain regions; irrespective of the seizure foci, epilepsy can lead to the development of anxiety whether impacting neurons within the epileptogenic zone or outside the seizure foci (253).

The HPA axis is a common link between epilepsy and anxiety (253–255). Stress is a common trigger for seizures, and anxiety can manifest during an acute seizure episode (250, 254). In general, the HPA axis becomes hyperactive in response to stress to drive an appropriate behavioral response.

Epilepsy can lead to hyperactivity of the HPA axis, leading to increased cortisol levels (253). In addition to acute increases in cortisol levels following a seizure, persistent HPA axis hyperactivity and increased circulating cortisol occur over time with increased seizure burden. This reduces seizure threshold and can further promote spontaneous recurrent seizure (253, 256). Adrenocorticotrophin hormone (ACTH) and corticosteroids have been used to treat certain epilepsies and infantile spasms, but due to the common underlying mechanism of seizures and anxiety, HPA axis regulation has become an interesting target for drug development that may have therapeutic potential for epilepsy, anxiety, and mood disorders (255, 256).

### 7.3 Increased risk of mortality

People with epilepsy carry more than two-fold higher risk for premature death than the general population; furthermore, patients with DRE are at a higher risk of premature death compared with patients who are seizure free (257). This underscores the fact that all seizures have an impact, no matter the frequency or duration of an individual event, and the cumulative impact over time can have dire consequences for patients and families. The most common causes of death in patients with epilepsy are SUDEP, injury, or suicide (257). This further underscores the urgency to reduce seizure burden (both frequency and duration of seizure episodes) in patients with DRE and to effectively manage comorbidities.

SUDEP is defined as sudden, unexpected, witnessed or unwitnessed, nontraumatic, and non-drowning death in patients with epilepsy with or without evidence of a seizure, and excluding documented status epilepticus ≥30 min in duration, in which postmortem examination does not reveal a structural or toxicological cause for death (258). Numerous studies have examined the risk factors associated with SUDEP (257, 259–261). Patients with DRE are at a higher risk of SUDEP than patients who are seizure free on ASM therapy, so achieving seizure control can reduce the risk of SUDEP (259–261). Additionally, SUDEP has been linked to ASM nonadherence and subtherapeutic concentrations of ASMs at the time of SUDEP, and improved seizure control, ASM polypharmacy, and vagus nerve stimulation (VNS) significantly reduce SUDEP risk (262–264). Other factors that significantly increase risk of SUDEP are generalized tonic–clonic seizure episode(s) during the last year, nocturnal seizures, living alone, and not sharing a bedroom (257, 259–261). For patients experiencing tonic–clonic seizures, the risk of SUDEP significantly increases as the frequency of these seizures increases (259–261). Patients who experience one to three generalized tonic–clonic seizures in one year are associated with a 22-fold increased risk of SUDEP, while patients who experience four to ten generalized tonic–clonic seizures at a 32-fold increased risk. Mitigating the risk of SUDEP is critical when treating patients with DRE, and it is important that patients and care partners understand these risks and how to reduce them (261, 265, 266).

Extremely concerning are the respiratory and cardiovascular comorbidities that contribute to SUDEP (208, 210, 265–268). Although not well understood, there have been attempts to gain insight into the sequence of events that trigger SUDEP (265, 266). The structural changes at the neuronal level discussed above also play a role in interrupting homeostatic maintenance in the peri-ictal period, with cellular changes in brain regions involved in autonomic control leading to E/I imbalance and loss of autonomic control, ultimately culminating with the cardiorespiratory dysfunction that is thought to underly SUDEP (208, 265, 267).

Due to the involvement of limbic structures in regulating the autonomic nervous system, alterations in autonomic control are more pronounced in TLE, especially when associated with focal-to-bilateral tonic–clonic seizures (265). However, due to the widespread structural and functional changes occurring in larger neuronal networks due to seizure, the accumulation of these changes can increase risk of SUDEP regardless of epileptogenic foci (265, 266). Several receptor systems have been implicated in SUDEP, including the catecholamine, adenosine, and serotonergic systems (208, 210, 265–268). Increased catecholamine release during seizures has been linked to myocardial damage (208). Catecholamine surge and strong sympathetic activation may explain the sudden onset of focal or generalized tonic–clonic seizure-dependent ventricular tachycardia and ventricular fibrillation (265). In line with this hypothesis, catecholamine-mediated activation of both beta-adrenoreceptors and alpha-adrenoreceptors is linked to increased Ca^2+^ release, coronary spasm, myocardial ischemia, platelet activation, and auto-oxidation, impacting cardiovascular health (266). In addition to catecholamine release, adenosine release is increased following seizure, and adenosine can accumulate with continued seizure, leading to spreading depolarization in the amygdala and brainstem and subsequent respiratory depression and respiratory collapse, contributing to the respiratory mechanisms associated with SUDEP (266–268). The serotonergic system also plays an important role in respiratory regulation and has been implicated in risk of SUDEP (265–267). Increased serotonergic activity is associated with seizure suppression; moreover, following seizures, patients with central apnea had reduced postictal serotonin compared with patients without central apnea (267, 269). It is thought that the blunted serotonergic activity following seizures leads to an impaired arousal response and to increased CO_2_ levels, which causes hypoventilation and respiratory collapse (265, 266).

Fighting for reduced seizure burden and improved seizure control is paramount for patients at risk for SUDEP. Several treatment strategies can significantly reduce a patient’s overall risk for SUDEP. The primary strategy is ensuring patients are well aware of risk factors associated with SUDEP and that they are educated about treatment options that reduce their overall risk is highly important (261, 265, 266, 270). Due to the significantly increased risk of SUDEP in patients who have even a single convulsive seizure, it is extremely important to continually attempt to reduce the overall number of generalized tonic–clonic seizures or focal-to-bilateral tonic–clonic seizures (261, 265, 266, 270). As such, the risk of SUDEP is reduced in patients on multiple ASMs and those who are adherent to their ASM regimen (261, 265, 266, 270). As nocturnal seizures are associated with a higher risk of SUDEP, nighttime monitoring is another treatment approach that significantly reduces the risk of SUDEP (261, 265, 266, 270). Wearable devices and monitors that alert to seizure or cardiorespiratory distress, as well as the use of safety pillows, are recommended for patients at high risk of SUDEP (222, 265). In addition, it is important for care partners to understand first aid, so that they can address hypoventilation and administer oxygen as needed (266, 270).

### 7.4 People with epilepsy and QoL

Patients and care partners can report disease progression and often express concern about what that means for themselves and their loved ones. Directly related to the pathophysiology discussed above, patients present with many concerns regarding how cognitive impairment will impact their concentration, ability to work, and ability to remember (271). Following disease progression, patients on more than three ASMs report more reductions in QoL than patients on fewer chronic daily medications (271). The perceived stigma becomes very socially isolating and burdensome and has significant impacts on social engagement, social cognition, and patients’ mental health (271, 272). In addition, daily living activities are impacted; patients can lose not only the ability to drive but also the ability to carry out typical daily activities, such as cleaning, climbing stairs, and engaging in hobbies or exercising, which further impacts mental and physical health (271). Treatment strategies that augment daily ASM treatment and mitigate concerns regarding the unpredictability of spontaneous, recurrent seizures can positively impact patients’ QoL by increasing social functioning and reducing seizure worry (273). It is critical from both a QoL and a long-term brain health perspective that no spontaneous, recurrent seizures are considered benign and that we continually work to reduce overall seizure burden in patients with DRE (214, 274).

## 8 Summary

### 8.1 Seizure timeline

In 2023, the American Academy of Neurology (AAN) published a call to action, stating that:

Neurologists are uniquely positioned to serve as specialists in brain health and to advance the newly evolving field of preventive neurology, which aims to identify individuals at high risk of brain disorders and other neurologic conditions and offer strategies to mitigate disease emergence or progression (204).

The question remains, how can we develop treatment strategies to reduce or eliminate the cascade of events following a seizure to prevent the establishment of seizure networks or reduce or prevent the ongoing progression of disease? Although we have yet to find a panacea that can alter epileptogenesis or the underlying mechanisms responsible for disease progression, understanding the temporal nature of events that initiate these processes is important to identify treatment approaches that directly interfere with the disease process.

**Figure 5:**
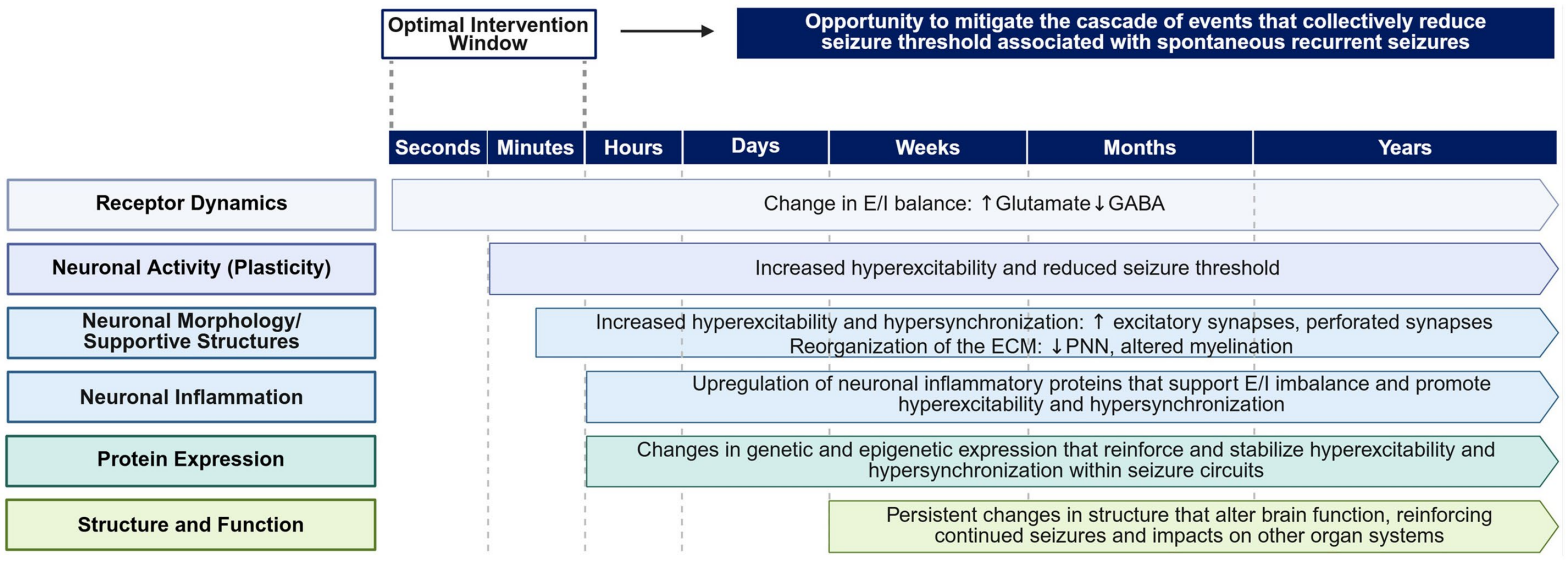
Seizure timeline: how seizures beget seizures. This timeline outlines the cascade of events initiated and reinforced by continued seizures that lead to pathological structural and functional changes that support disease progression. This is an attempt to synthesize the temporal changes that occur with a seizure that underly increases in seizure frequency, duration, and severity, and establishes the basis of why “seizures beget seizures.” Figure created in BioRender. ECM, extracellular matrix; E/I, excitatory/inhibitory; GABA, gamma-aminobutyric acid; PNN, perineuronal net.

To gain an understanding of the temporal progression of illness, we have created a seizure timeline that outlines the temporal nature of seizures and specifically the timing of the cascade of neurological events that occur following an acute seizure and are involved in epileptogenesis and ongoing seizure progression. Figure 5 summarizes the cascade of neurochemical, structural, neuroinflammatory, genetic, and epigenetic events following seizure that impact overall brain function and support the reinforcement of seizure activity characteristic of DRE. As Vezzani et al. (74) point out, the pathophysiological mechanisms that underly the onset and recurrence of epileptic seizures and comorbidities are still mostly unresolved. However, distilling what we know about seizure processes and the cascade of events that leads to disease progression is an important first step in determining what we can do with our current treatment strategies in order to maximize the reduction in seizure frequency, duration, and severity for each patient.

### 8.2 Retraining seizure networks

The processes underlying the function of neuronal circuits are dynamic. Seizure activity can overwhelm normal function and entrain circuits to be hyperexcitable and hypersynchronous, as seen in individuals with epilepsy. The underlying neurobiology driving increased neuronal excitation and reduced inhibition has led to the development of over 30 ASMs that aim to provide E/I balance and achieve seizure freedom (3). Yet, approximately 40% of patients with epilepsy develop DRE and continue to suffer with the consequences associated with ongoing seizures (4). Can seizure networks be retrained to promote longer-term reduction in associated burden? Emerging data with responsive neurostimulation (RNS) devices suggest this is possible. Designed to continuously monitor neural activity, RNS devices deliver electrical stimulation through electrodes in response to specific patterns of brain activity that precede a seizure, with the aim of disrupting seizure onset and reducing the frequency, duration, and severity of seizures (275). RNS in response to these epileptiform discharges disrupts epileptogenic excitatory networks and increases inhibitory activity patterns, thereby promoting healthy network reorganization and increasing seizure threshold (275, 276). The long-term effects of retraining these neuronal networks are demonstrated by continued seizure burden reduction over 9 years with RNS utilization (8). Electrical stimulation that modulates the GABAergic system is a critical component of the underlying mechanism of the antiseizure effects of RNS (9, 276). RNS stimulation and modulation of the GABAergic system have been shown to be involved in seizure cessation and prevention, which strengthens the inhibitory networks that increase seizure threshold (9, 276).

Evidence from the RNS literature suggests that, over time, seizure-induced physiological changes result in increased inhibitory network influences that can render the brain less susceptible to seizure. Based on receptor dynamics and the cascade of events following a seizure, timely and effective seizure management can increase inhibitory drive to alter or disrupt the seizure cascade and limit its overall impact on E/I imbalance. Furthermore, by increasing the inhibitory drive associated with each seizure episode, neuronal networks may be retrained in a similar manner to that observed with RNS, leading to E/I rebalancing and a potential increase in the seizure threshold. As novel pharmacotherapies targeting the underlying cause of seizures continue to be developed, it may become possible to leverage the dynamic properties of neural networks to pharmacologically retrain pathological seizure circuits toward a more balanced state and restore proper inhibitory control.

## 9 Conclusion

Understanding the seizure timeline and the cascade of neurophysiological changes that lead to spontaneous, recurrent seizures is critically important for managing patients with DRE. Equally important is understanding how to identify patients with DRE who could benefit from a comprehensive treatment approach that aims to maximize the reduction in seizure frequency, duration, and severity. Risk factors can suggest that a patient is dealing with frequent episodes of seizures, and augmenting their chronic ASM treatment with neuromodulation implantables, pro re nata immediate-use seizure medications, or surgery could significantly benefit patient outcomes and QoL.

With the emphasis that AAN has put on brain health and creating mitigation strategies to prevent the progression of disease, a comprehensive treatment approach to combat spontaneous, recurrent, frequent episodes of seizures may improve the long-term clinical outcomes and QoL of patients and care partners dealing with DRE.
